# Vitamin D deficiency and cardiovascular risk in type 2 diabetes population

**DOI:** 10.1515/biol-2021-0050

**Published:** 2021-05-10

**Authors:** Sushant Pokhrel, Nisha Giri, Rakesh Pokhrel, Bashu Dev Pardhe, Anit Lamichhane, Abhisek Chaudhary, Mahendra Prasad Bhatt

**Affiliations:** Department of Laboratory Medicine, Manmohan Memorial Institute of Health Sciences, P. O. Box No. 15201, Kathmandu, Nepal; Department of Genetics, National academy of Medical Sciences, Bir Hospital, Kathmandu, Nepal; Department of Biochemistry, Institute of Medicine, Maharajgunj Medical Campus, Kathmandu, Nepal; Department of Life Science and Biochemical Engineering, Sun Moon University, Asan-Si, Chumgnam, South Korea; Department of Clinical Pathology, Modern Diagnostic Laboratory and Research Center, Kathmandu, Nepal

**Keywords:** vitamin D, dyslipidemia, glycemic control, type 2 diabetes mellitus

## Abstract

This study aims to assess vitamin D deficiency-induced dyslipidemia and cardiovascular disease (CVD) risk in poor glycemic control among type 2 diabetes mellitus (T2DM) patients. This study was carried out among 455 T2DM patients involving poor glycemic control (*n* = 247) and good glycemic control (*n* = 208). Fasting plasma glucose (FPG) and HbA_1_c were measured to assess glycemic control. Cardiac risk ratio, atherogenic index plasma, and atherogenic coefficient were calculated to assess and compare the CVD risk in different groups. Patients with poor control had a significantly higher level of total cholesterol (TC), triglyceride (TG), and non-high-density lipoprotein lipase cholesterol (non-HDL-C), atherogenic variables, and lower level of high-density lipoprotein lipase cholesterol (HDL-C) as compared to patients with good glycemic control. We also observed significant negative correlation of vitamin D with lipid markers and atherogenic variables in poor glycemic control diabetic population. The serum vitamin D levels were inversely associated with HbA_1_c, FPG, TG, TC, and non-HDL-C. Furthermore, hypercholesterolemia, hypertriglyceridemia, and elevated non-HDL-C were the independent risks in hypovitaminosis D population. Vitamin D deficiency in poor glycemic control is likely to develop dyslipidemia as compared to vitamin D insufficient and sufficient groups. Thus, vitamin D supplementation and an increase in exposure to sunlight may reduce the risk of cardiovascular complications in diabetes.

## 1 Background

Diabetes mellitus (DM) is a chronic metabolic disease manifested by elevated glucose level in plasma either because of insulin deficiency (type 1 DM) or insulin resistance (type 2 DM) [1]. Diabetes is a primary factor for dyslipidemia which promotes cardiovascular complications. Dyslipidemia is a metabolic abnormality leading to increase plasma concentration of total cholesterol (TC) or triglyceride (TG) or a decrease in high-density lipoprotein cholesterol (HDL-C) [2]. This hypercholesterolemia and hypertriglyceridemia are because of disruption of the tightly regulated process of cholesterol, TG, and their derivatives which is because of persistent hyperglycemia in diabetes [3].

Primarily, vitamin D acts as a hormone, which is known to be essential for calcium homeostasis and bone growth. Along with the skeletal requirement, vitamin D receptors are also present in other tissues with vital roles such as insulin secretion, immune mechanism, gene expression, and cardiovascular protection [4]. Moreover, vitamin D plays a significant role in minimizing chronic metabolic syndromes such as type 2 DM (T2DM) and cardiovascular diseases (CVDs) [5]. Active form of vitamin D (1,25-dihydroxy vitamin D) induces insulin responsiveness for glucose transport by stimulating the expression of the insulin receptor in peripheral tissue [6]. Vitamin D and its metabolite downregulate the serum lipid by lipogenesis, but the mechanism is still unclear. However, vitamin D activates lipoprotein lipase activity in adipocytes, resulting in a reduction of TG-enriched lipoprotein from the blood, thus reducing the risk of development of CVD [7]. Vitamin D receptors are present in the myocardial and vascular cells suggesting the involvement of vitamin D-mediated effect in the development of CVD [8]. Vitamin D has shown an effect in the lipid profile of the diabetic patient in some clinical research [5, 9]. Thus, this study aims to find the relationship between glycemic control and vitamin D in diabetes population. In addition, this study aims to find out the association of vitamin D deficiency and increase CVD risk in poor glycemic control T2DM Nepalese population.

## 2 Materials and methods

This descriptive cross-sectional study was carried out for a period of 10 months (March 2019 to December 2019) in Modern Diagnostic Laboratory and Research Center (MDRC), Kathmandu, Nepal in collaboration with Manmohan Memorial Institute of Health Sciences (MMIHS), Kathmandu, Nepal.

### 2.1 Inclusion and exclusion criteria

Among 568 patients already diagnosed with T2DM attending the MDRC, 113 patients with chronic metabolic disease, pregnancy, treatment on vitamin D supplementation, and lowering agent were excluded from the study. Therefore, a total of 455 patients under hyperglycemic treatment were included in the study.


**Informed consent:** Informed consent has been obtained from all the individuals included in this study.
**Ethical approval:** The research related to human use has been complied with all the relevant national regulations and institutional policies and in accordance with the tenets of the Helsinki Declaration, and has been approved by the Institutional Review Committee of Manmohan Memorial Institute of Health Sciences, Kathmandu, Nepal (Approval registration number – MMIHS IRC 449).

### 2.2 Experimental protocol

Anthropometric variables and clinical characteristics were recorded using a standard questionnaire. Height was measured by standing erect with barefoot using a wall scale meter. The weight of the patient was measured with barefoot and minimal clothing using the standard digital weighing machine. Body mass index (BMI) was calculated and expressed in kg/m^2^. The presence of hypertension was considered among the patient with a prior diagnosis of hypertension by a clinician and/or systolic blood pressure ≥140 mm Hg and/or diastolic blood pressure ≥90 mm Hg and/or under treatment with antihypertensive drugs [10]. Patient with a history of regular alcohol use of at least two drinks per day was considered as “alcohol consumption” and regular smoking were classified as “presence of smoking.”

### 2.3 Biochemical analysis

Blood sample with fasting of 8–12 hours was collected by venipuncture in a PET tubes containing tripotassium EDTA as anticoagulant for whole blood (Henso vacuum tube, China), sodium fluoride as anticoagulant for plasma (Henso vacuum tube, China) gel-tube and clot activator (Henso vacuum tube, China) for serum. The biochemical parameters such as fasting plasma glucose (FPG) and lipid profile were measured by fully automated Dimension RxL Max integrated chemistry analyzer (Siemens, Munich, Germany). Vitamin D was measured by a fully automated Advia centaur XP immunoassay (Siemens, Munich, Germany). HbA_1_c was measured by H9 fully automated High Performance Liquid Chromatography HbA_1_c analyzer (Lifotronic, Shenzhen, China). All the biochemical parameters were expressed in mmol/L, while HbA_1_c in percentage (%) and vitamin D in ng/mL. Internal quality control was performed daily, while external quality control was performed quarterly for validation of the test. The intra- and inter-assay coefficients of variation (CVs) for vitamin D were 4.7 and 5.1%, respectively, while for HbA_1_c were 4.9 and 6.2%, respectively, and the CVs for FPG, TC, TG, and HDL-C were <6% (intra-assay) and 7% (inter-assay).

The atherogenic indices were calculated using the formula described in previous study [11] as:

\begin{array}{c}\text{Cardiac}\hspace{.5em}\text{risk}\hspace{.5em}\text{ratio}\hspace{.5em}(\text{CRR})=\text{TC}/\text{HDL-C}\\ \text{Atherogenic}\hspace{.5em}\text{coefficient}\hspace{.5em}(\text{AC})=(\text{TC-HDL-C})/\text{HDL-C}\\ \text{Atherogenic}\hspace{.5em}\text{index}\hspace{.5em}\text{of}\hspace{.5em}\text{plasma}\hspace{.5em}(\text{AIP})=\hspace{.25em}\log (\text{TG/HDL-C}).\end{array}]

FPG, HbA_1_c, vitamin D, lipid profile, and atherogenic indices were measured in both the groups to find the associations and correlations. Furthermore, vitamin D level was used to categorize patients as deficient, insufficient, and sufficient groups according to NKF/KDOQI guideline [12]. Serum vitamin D level with 30–100 ng/mL was considered to be sufficient, while less than 30 ng/mL as hypovitaminosis D. The patients with HbA_1_c <7% were categorized as good glycemic control and those with HbA_1_c ≥7% were considered poor glycemic control. Dyslipidemia was defined as hypercholesterolemia >200 mg/dL (>5.2 mmol/L), hypertriglyceridemia 150 mg/dL (>1.7 mmol/L), and reduced HDL-C <40 mg/dL (<1.03 mmol/L) in men and <45 mg/dL (<1.2 mmol/L) in women.

### 2.4 Statistical analysis

All the data were collected and analyzed by SPSS version 20 (IBM corporation, Armonk, NY, USA). The normal distribution of the variables was analyzed by Shapiro–Wilk test. Categorical variables were presented as numbers and compared using chi-square test. Continuous variables were shown as median (25th–75th percentile) and compared using Mann–Whitney *U*-test. Correlation with vitamin D with different biochemical variables was performed by Spearman’s correlation. Multiple linear regression analysis was used to estimate the association between serum 25(OH) D and biochemical variables. Further, logistic regression analysis was carried out to evaluate the risk of dyslipidemia in hypovitaminosis D patients. A *p*-value of less than 0.05 was considered statistically significant.

## 3 Results

### 3.1 General characteristics

Among the 455 T2DM patients, the median serum vitamin D level of the study population was 18.56 ng/mL and *p*_25_ and *p*_75_ were 14.36 and 26.35 ng/mL. In total, 83.3% of the study participants were with hypovitaminosis D (vitamin D <30 ng/mL). In this study, 247 participants were with poor glycemic control while 208 were with good glycemic control. The median age of the study population was 56 years ranging from 26 to 93 years.

Sociodemographic and anthropometric characteristics of participants are summarized in Table 1. Duration of diabetes was significantly higher in poor glycemic control in comparison to good glycemic control diabetes population (*p* = 0.002). In addition, the presence of hypertension (*p* = 0.024) and alcohol consumption (*p* = 0.011) was significantly higher in the poor glycemic control diabetes population.

**Table 1:** Comparison of sociodemographic and anthropometric variables between good and poor glycemic control diabetic population

Variables	Good glycemic control	Poor glycemic control	*p*
Age (years)	55 (46–64)	57 (48–66)	0.171^a^
**Sex**			
Male	118	119	0.074^b^
Female	90	128	
BMI (kg/m^2^)	21.02 (18.69–24.29)	21.36 (19.45–24.15)	0.115^a^
Duration of diabetes (years)	7 (4–9)	9 (7–13)	**0.002** a
Presence of smoking habit	28	37	0.688^b^
Presence of hypertension	64	102	**0.024** b
Alcohol consumption	45	80	**0.011** b

BMI – body mass index.

*p*-value indicates the level of significance.

Non-normally distributed variables are presented as Median (*p*_25_–*p*_75_), while other variables are presented in numbers.

a Mann–Whitney *U*-test to analyze the non-normally distributed variables.

b Chi-square test used to analyze two categorical variables.

Bold values indicates the *p* < 0.05.

### 3.2 Association of biochemical parameters and atherogenic variables with glycemic control

FPG (*p* < 0.001), TC (*p* < 0.001), TG (*p* < 0.001), and non-HDL-C (*p* < 0.001) were significantly increased in patients with poor glycemic control in comparison to good glycemic control, while the levels of vitamin D (*p* = 0.031) and HDL-C (*p* = 0.019) were significantly higher in patients with good glycemic control (Table 2). Moreover, we observed that the good glycemic control group of the diabetes population had a significantly lower level of atherogenic measures cardiac risk ratio (CRR; *p* < 0.001), atherogenic coefficient (AC; *p* < 0.001), and atherogenic index plasma (AIP; *p* < 0.001) (Table 2).

**Table 2:** Comparison of the biochemical parameter and atherogenic variables between good and poor glycemic control diabetic population

Variables	Good glycemic control (*n* = 208)	Poor glycemic control (*n* = 247)	*p*
FPG (mmol/L)	6.806 (6.25–7.44)	7.78 (6.72–9.89)	<0.001
Vitamin D (ng/mL)	18.71 (15.40–26.32)	17.89 (12.85–26.57)	0.031
TC (mmol/L)	3.85 (3.31–4.58)	4.68 (3.78–5.46)	<0.001
TG (mmol/L)	1.52 (1.1–1.99)	1.98 (1.39–2.98)	<0.001
HDL-C (mmol/L)	1.08 (1.03–1.19)	1.03 (0.91–1.19)	0.019
Non-HDL-C (mmol/L)	2.84 (2.22–3.44)	3.59 (2.77–4.44)	<0.001
CRR	3.59 (3.02–4.32)	4.37 (3.62–5.27)	<0.001
AC	2.59 (2.02–3.32)	3.37 (2.62–4.27)	<0.001
AIP	0.51 (0.34–0.63)	0.63 (0.45–0.85)	<0.001

FPG – fasting plasma glucose; TC – total cholesterol; TG – triglyceride; HDL-C – high-density lipoprotein cholesterol; non-HDL-C – non-high density lipoprotein cholesterol; CRR – cardiac risk ratio; AC – atherogenic coefficient; AIP – atherogenic index plasma.

Mann–Whitney *U*-test has been employed to analyze the non-normally distributed variables.

*p*-value indicates the level of significance.

All the variables are presented as Median (*p*_25_–*p*_75_).

### 3.3 Association of vitamin D and lipid profile

Multiple regression analysis was used to assess the associations between vitamin D concentration and serum lipids, HbA_1_c, and FPG. Vitamin D concentration was negatively associated with HbA_1_c, FPG, TC, TG, and non-HDL-C after adjusting for age, sex, BMI, and duration of diabetes. Each 1 ng/mL increase in serum vitamin D was associated with a decrease of 0.097% in HbA_1_c, 0.119 mmol/L in FPG, 0.160 mmol/L in TC, 0.201 mmol/L in TG, and 0.166 mmol/L in non-HDL-C, whereas there was no significant association between HDL-C and vitamin D (Table 3).

**Table 3:** Multiple linear regression analysis for vitamin D and lipid profile

Variable	β-coefficient	*p*
HbA_1_c (%)	−0.097	**0.039**
FPG (mmol/L)	−0.119	**0.011**
TC (mmol/L)	−0.160	**0.001**
TG (mmol/L)	−0.201	**<0.001**
HDL-C (mmol/L)	0.009	0.577
Non-HDL-C (mmol/L)	−0.166	**0.001**

FPG – fasting plasma glucose; TC – total cholesterol; TG – triglyceride; HDL-C – high-density lipoprotein cholesterol; non-HDL-C – non-high density lipoprotein cholesterol.

*p*-value indicates the level of significance.

Bold values indicates the *p* < 0.05.

### 3.4 Correlation of vitamin D and serum lipid markers in the poor and good glycemic control

**Figure 1:**
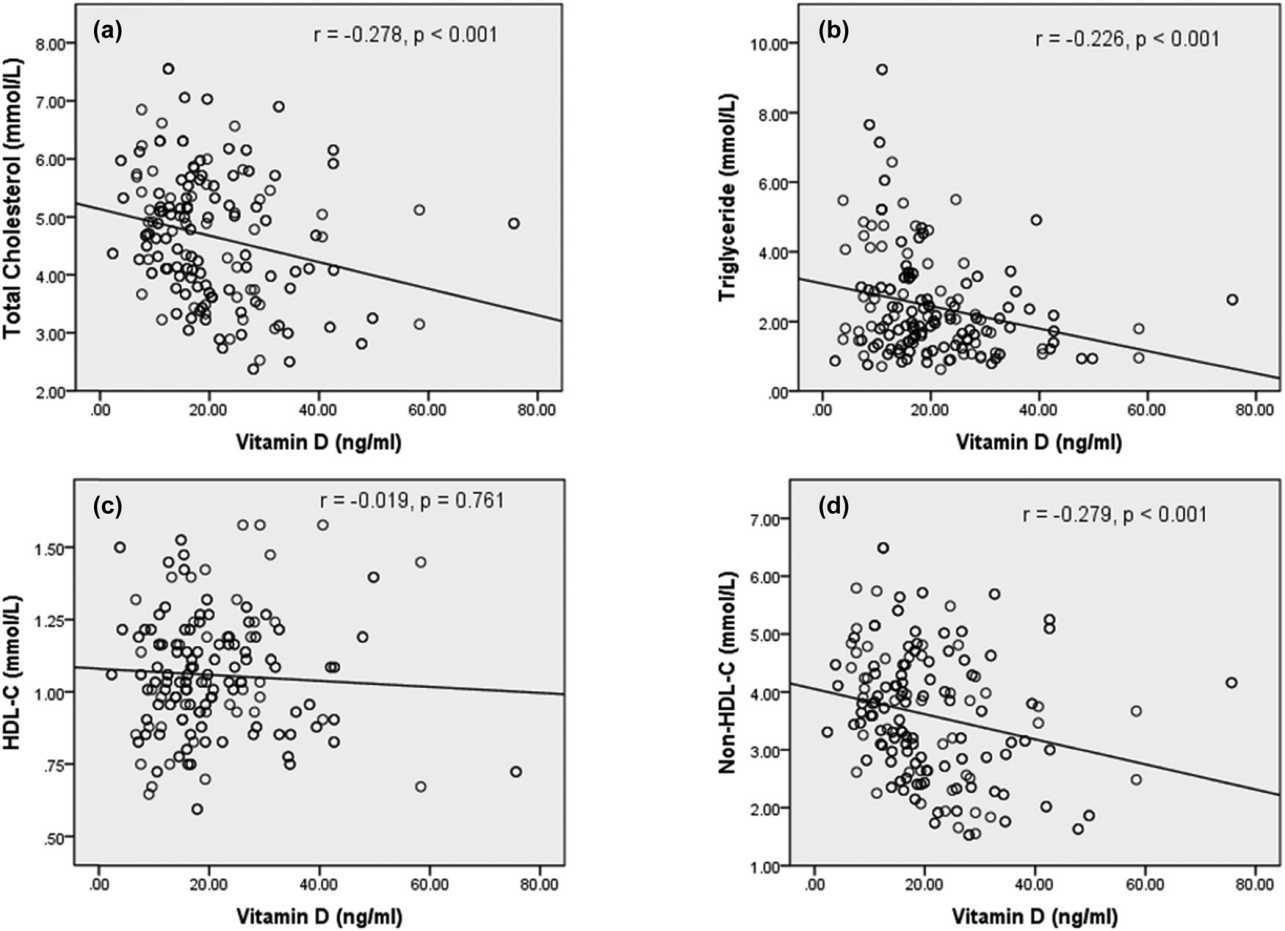
Correlation between vitamin D and serum lipid markers in the poor glycemic control diabetes population. (a) Correlation between total cholesterol and vitamin D; (b) correlation between triglyceride and vitamin D; (c) correlation between HDL-C and vitamin D; (d) correlation between non-HDL-C and vitamin D. Abbreviations: HDL-C – high-density lipoprotein cholesterol; non-HDL-C – non-high-density lipoprotein cholesterol. Note: *r* denotes the correlation coefficient and *p*-value indicates a level of significance.

**Figure 2:**
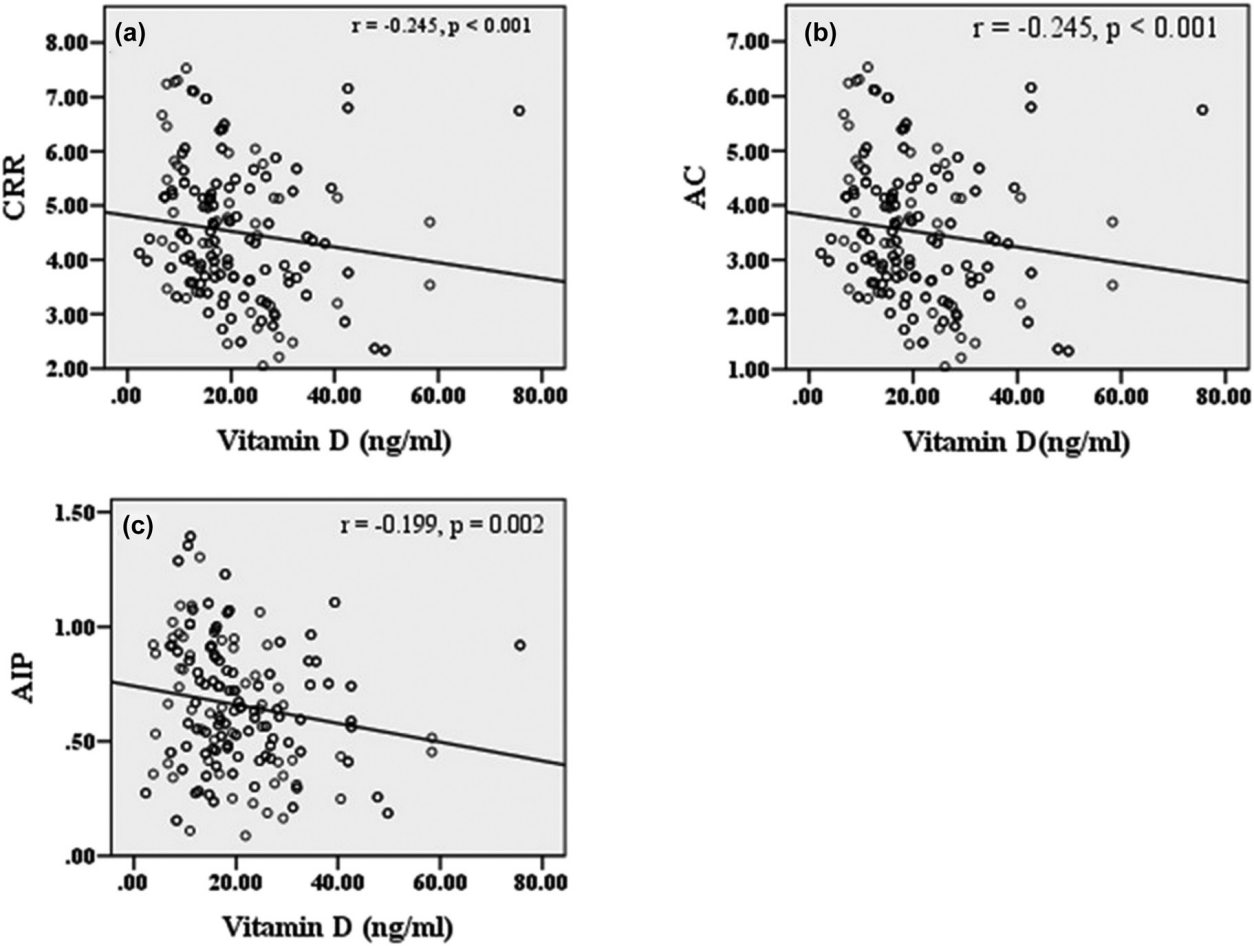
Correlation between vitamin D and atherogenic variables in the poor glycemic control diabetes population. (a) Correlation between CRR and vitamin D; (b) correlation between AC and vitamin D; (c) correlation between AIP and vitamin D. Abbreviations: CRR – cardiac risk ratio; AC – atherogenic coefficient; AIP – atherogenic index plasma. Note: *r* denotes the correlation coefficient and *p*-value indicates a level of significance.

**Figure 3:**
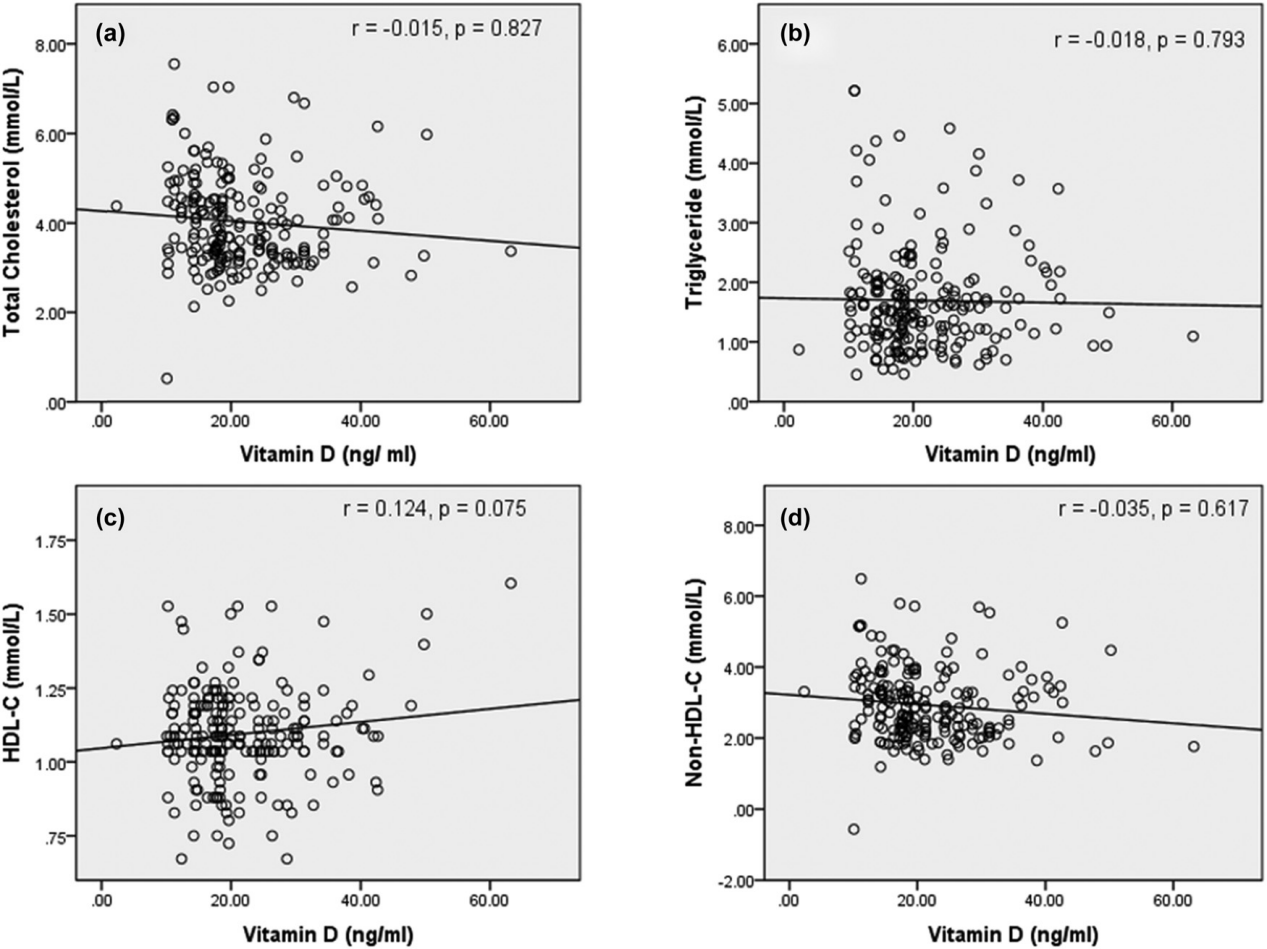
Correlation between vitamin D and serum lipid markers in the good glycemic control diabetes population. (a) Correlation between total cholesterol and vitamin D; (b) correlation between triglyceride and vitamin D; (c) correlation between HDL-C and vitamin D; (d) correlation between non-HDL-C and vitamin D. Abbreviations: HDL-C – high-density lipoprotein cholesterol; non-HDL-C – non-high density lipoprotein cholesterol. Note: *r* denotes the correlation coefficient and *p*-value indicates a level of significance.

**Figure 4:**
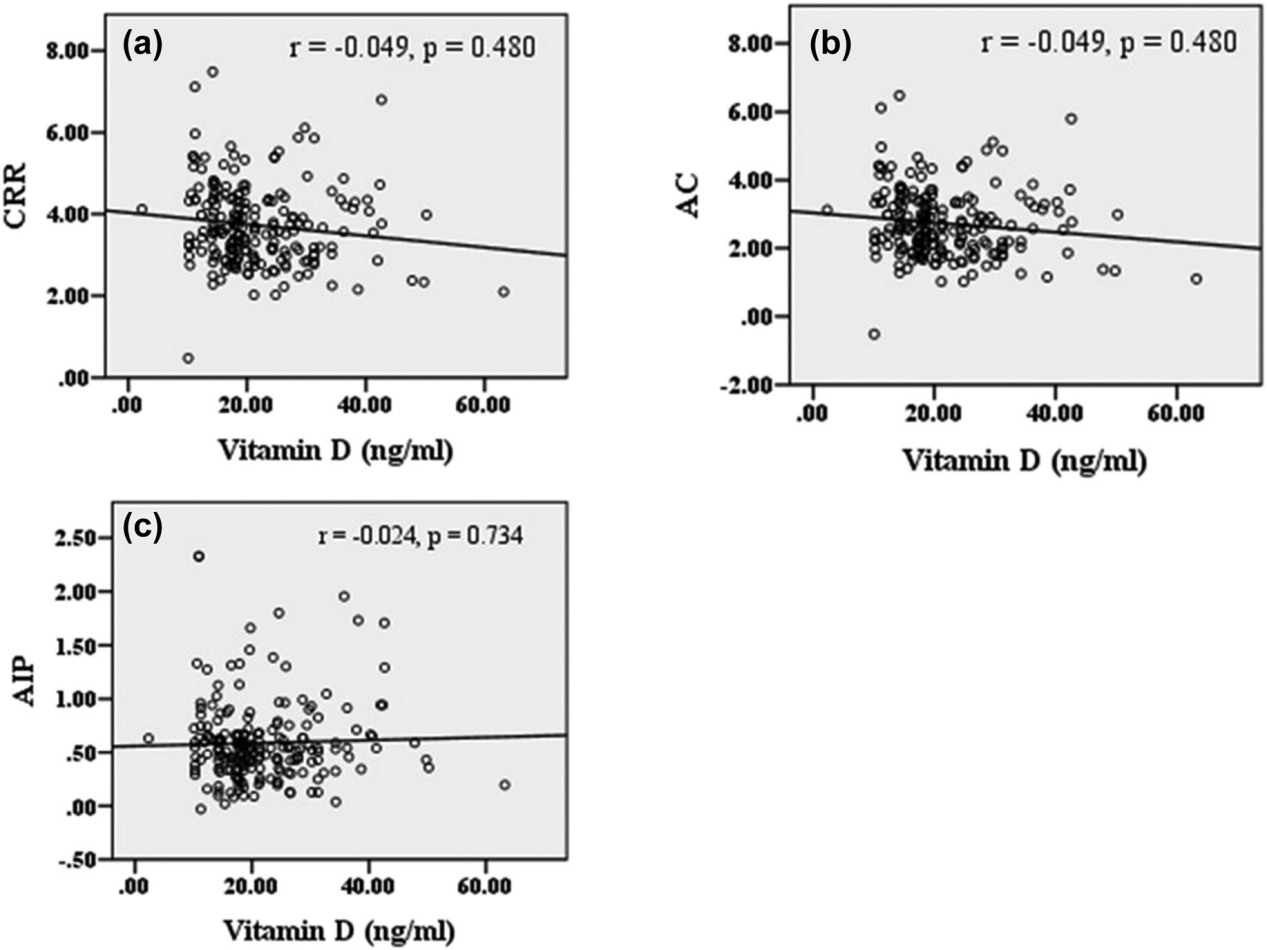
Correlation between vitamin D and atherogenic variables in the good glycemic control diabetes population. (a) Correlation between CRR and vitamin D; (b) correlation between AC and vitamin D; (c) correlation between AIP and vitamin D. Abbreviations: CRR – cardiac risk ratio; AC – atherogenic coefficient; AIP – atherogenic index plasma. Note: *r* denotes the correlation coefficient and *p*-value indicates a level of significance.

Spearman’s rank correlation coefficient was performed for the association between vitamin D with serum lipids and atherogenic variables in poor and good glycemic control. Vitamin D concentration showed a negative association with TC (*r* = −0.278, *p* < 0.001), TG (*r* = −0.226, *p* < 0.001), and non-HDL-C (*r* = −0.279, *p* < 0.001) (Figure 1). Figure 2 demonstrates the correlation between atherogenic variables with vitamin D in poor glycemic control diabetes patients. Atherogenic variables, i.e., CRR (*r* = −0.245, *p* < 0.001), AC (*r* = −0.245, *p* < 0.001), and AIP (*r* = −0.199, *p* = 0.002), showed a significantly negative correlation with vitamin D levels. However, there was no significant correlation between vitamin D with TC (*r* = −0.015, *p* = 0.827), TG (*r* = −0.018, *p* = 0.793), HDL-C (*r* = 0.124, *p* = 0.075), non-HDL-C (*r* = −0.035, *p* = 0.617), CRR (*r* = −0.049, *p* = 0.48), AC (*r* = −0.049, *p* = 0.48), and AIP (*r* = −0.024, *p* = 0.734) in good glycemic control diabetes patients (Figures 3 and 4).

### 3.5 Risk analysis of dyslipidemia in hypovitaminosis D-deficient diabetes patient

Unadjusted OR and age, sex, BMI, duration of diabetes, presence of hypertension, smoking habit, and alcohol consumption adjusted OR for dyslipidemia, higher HbA_1_c, and hyperglycemia are presented in Table 4. Among all the independent variables, hypercholesterolemia (AOR = 1.37, 95% CI: 1.007–2.994), hypertriglyceridemia (AOR = 1.836, 95% CI: 1.172–2.876), and elevated non-HDL-C (AOR = 1.838, 95% CI: 1.176–2.872) were the independent risks in hypovitaminosis D population.

**Table 4:** Multivariate logistic regression analysis of poor glycemic control and dyslipidemia in hypovitaminosis D population

Variable	COR [95% CI]	*p*	AOR [95% CI]	*p*
Higher HbA_1_c	0.978 [0.649–1.474]	0.914	1.316 [0.827–2.094]	0.246
Higher FPG	1.735 [0.836–3.602]	0.139	1.938 [0.912–4.117]	0.085
Hypertriglyceridemia	1.671 [1.099–2.543]	**0.016**	1.836 [1.172–2.876]	**0.008**
Hypercholesterolemia	1.479 [0.882–2.479]	0.138	1.737 [1.007–2.994]	**0.047**
Reduced HDL-C	1.123 [0.729–1.731]	0.599	1.184 [0.758–1.850]	0.458
Higher Non-HDL-C	1.563 [1.036–2.359]	**0.033**	1.838 [1.176–2.872]	**0.007**

FPG – fasting plasma glucose; HDL-C – high-density lipoprotein cholesterol; non-HDL-C – non-high density lipoprotein cholesterol; COR – crude odd ratio; AOR – adjusted odd ratio.

*p*-value indicates the level of significance.

Bold values indicates the *p* < 0.05.

## 4 Discussion

The study found that 83.3% of the patients involved in the study were hypovitaminosis D. The study conducted by Bhatta et al. in Nepal reported the prevalence of 73.68% vitamin D deficiency which is similar to our study [13]. The prevalence of vitamin D deficiency in the diabetic population was found consistent in a previous study conducted in Saudi Arabia which was 76.6% [14]. The high prevalence of vitamin D deficiency in the Nepalese population might be because of low diet content vitamin D, as well as other risk factors such as skin pigmentation, wearing of well-covering clothes, and unawareness of diet content [12]. Estimating this scenario, in this study, we measured the association of vitamin D with the state of glycemic control along with lipid profile and atherogenic variables among the Nepalese T2DM population. There were no prior studies on the prevalence of hypovitaminosis D in T2DM and its association with poor glycemic control and cardiovascular risk in Nepal. Our study also showed that poor glycemic control had significantly increased level of lipid markers in comparison to good glycemic control diabetes population. This study has also revealed that higher level atherogenic variables significantly associated with poor glycemic control as indicated by a higher level of HbA_1_c. Taken together, the study demonstrated that patients with poor diabetic controls are at the higher risk of developing atherosclerosis and cardiovascular morbidity.

As described in previous studies [15–17], a relatively higher level of vitamin D is significantly associated with good glycemic control diabetes population which is found to be similar to our study. We also found that a decrease in 1 ng/mL in vitamin D was associated with an increase in 0.097% in HbA_1_c and 0.119 mmol/L in FPG. Similar to our study, the study by Yang et al. showed a negative association with FPG [18]. In contrast to our findings, the study by Saedisomeolia et al. showed no significant association with the level of HbA_1_c [19]. Some researchers suggest that specific vitamin D receptors are present in pancreatic β-cells, which have had a direct effect on insulin secretion and regulate glucose homeostasis in T2DM [20], while a study explained that vitamin D enhances insulin responsiveness for glucose transport in skeletal muscle by the expression of insulin receptors [6]. In addition, the presence of vitamin D response element in the human insulin gene promoter gets activated by the active form of vitamin D, which is responsible for the expression of insulin to maintain glucose homeostasis [21].

In our findings, data revealed that vitamin D had an inverse, significant association with TC (β-coefficient = −0.160) and TG (β-coefficient = −0.201) after adjusting the confounding variables, while findings of Saedisomeolia et al. [19] showed a negative insignificant association with TC and TG. Similar to our findings, Saedisomeolia et al. showed a positive association with HDL-C. The study by Chiu et al. showed that vitamin D had a significant association with TC, but no significant association with TG [22]. The findings of Ge et al. in China [23] showed that vitamin D had no significant association with TC and TG, but significant association with HDL-C, while the studies by Rolim et al. in Brazil [24] and Yang et al. in China [18] showed a significant negative association with TC, and the study by Yu et al. in Korea [16] showed a significant association with TG. Further, we measured the correlation to find the relationship between vitamin D and lipid markers in poor and good glycemic control population. There was a significant negative correlation of TC, TG, and non-HDL-C with vitamin D in poor diabetic control patients. However, there was no significant relationship with HDL-C in poor glycemic control population. Thus, our findings suggest that vitamin D deficient subjects are prone to develop dyslipidemia in poor control of T2DM.

**Figure 5:**
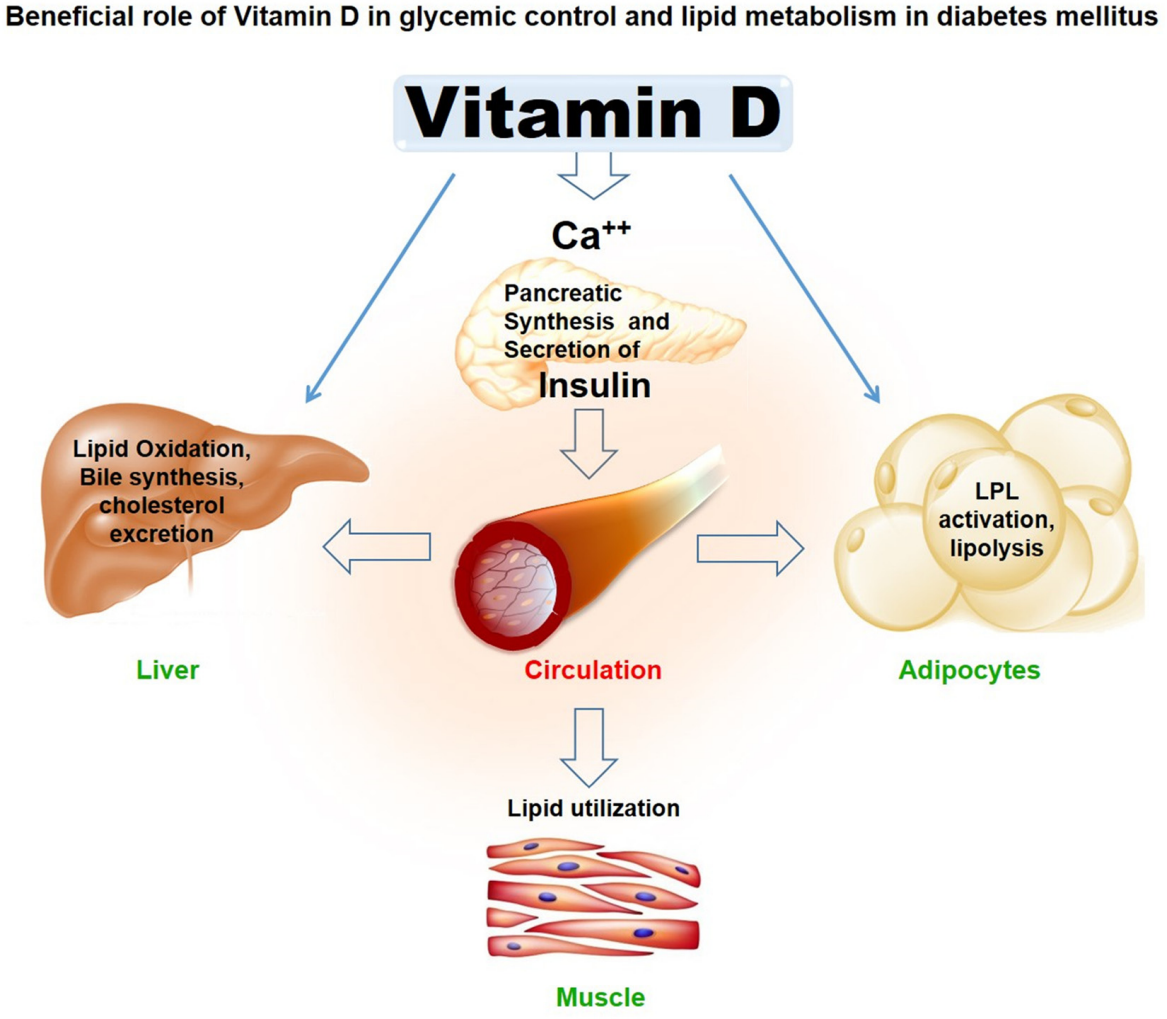
Schematic role of vitamin D in insulin synthesis and secretion, and lipid metabolism in diabetic patients. Vitamin D directly or indirectly i.e. via calcium signaling enhances insulin synthesis and secretion which has impact on glycemic control in circulation enhance lipoprotein lipase activation that inhibit lipogenesis or increase lipolysis in adipose tissue; lipid utilization in muscles; and enhance lipid oxidation, bile synthesis, and cholesterol excretion by liver.

Although the effect of vitamin D on lipid metabolism is poorly understood, it is found to be beneficial in glycemic control and lipid metabolism (Figure 5). Vitamin D affects the absorption of the calcium level that influences the synthesis and release of pancreatic insulin. The previous study suggested that higher concentration of calcium decreased the cholesterol level by the action of bile acid secretion [25]. Circulating cholesterol level affects the action of vitamin D on the transcription of vitamin D receptor and insulin-induced gene-2 (Insig-2), which downregulates the enzyme of cholesterol synthesis [26]. Diabetic population with hypovitaminosis D may suppress the signals of vitamin D receptor and result in the development of foam cells, subsequently increasing the cholesterol level in blood, which contribute to atherogenesis leading to CVDs [27]. Similarly, hepatic TG formation and secretion may be reduced by increased level of calcium [28]. The activity of lipoprotein lipase is enhanced by vitamin D which affects lipid metabolism [29]. Some studies have come up with a logic that vitamin D affects the β-cell function and insulin sensitivity which may be a potential pathogenic mechanism for the development of dyslipidemia in vitamin D deficiency, as the dyslipidemia is closely related to the insulin sensitivity [30].

Non-HDL-C represents all plasma lipid components including chylomicron, VLDL-C, IDL-C, LDL-C, except HDL-C, which is an excellent marker for CVDs risk and lipid disorder [31]. It can be measured in the non-fasting patient sample and the patient with a higher level of TGs (>400 mg/dL) [32]. In the present study, we found that non-HDL-C was negatively associated with the serum vitamin D concentration (β-coefficient = −0.166, *p* = 0.001). The notable finding in our study is that non-HDL-C in poor diabetic control has been negatively correlated (*r* = −0.228) with vitamin D at a significant level (*p* = 0.001) (Figure 1d), while vitamin D does not show a relationship with good control of diabetes (Figure 3d). The study of Sriram et al. in the USA also suggests an inverse correlation between vitamin D and non-HDL-C [33], which supports our findings. Long-term cardiovascular risk is predicted by the increasing concentration of non-HDL-C in the T2DM patient [32]. Several studies suggest that 1 mg/dL increase in non-HDL-C increases the risk of cardiovascular morbidity by 5% [34]. As likely to the above findings, this finding also supports that vitamin D-deficient poor control diabetic population is at an increased risk of cardiovascular morbidity.

Atherogenic indices are also the predictive value of cardiovascular risk. We analyzed the correlation of an atherogenic variable with vitamin D in both poor and good glycemic control diabetic population. Atherogenic variables showed a significant inverse relation with vitamin D in poor glycemic control, which indicates hypovitaminosis D as a potential atherogenic trigger for the development of CVDs. In our findings, AIP was significantly higher in vitamin D-deficient diabetic population with poor glycemic and negatively correlated with serum vitamin D (*r* = −0.199, *p* = 0.002) (Figure 2c). An increase in AIP affects insulin secretion and β-cell dysfunction and causes poor glycemic control in T2DM patients(46). We found that CRR (*r* = −0.245, *p* < 0.001) (Figure 2a) and AC (*r* = −0.245, *p* < 0.001) (Figure 2b) negatively correlated with serum vitamin D at a significant level, which are the major determinant to predict the cardiovascular risk. However, the relationship between vitamin D levels with lipid markers and atherogenic variables among good glycemic control of the diabetes population evaluated in this study was not significant. These findings together strongly suggest that vitamin D-deficient poor glycemic control patients of type 2 diabetes are prone to cardiovascular morbidity.

In addition, our previous studies provide further insights into further research in hyperglycemia-induced vasculopathy, and their prevention strategies can be well studied using human endothelial cells and in diabetic mice [35, 36]. Thus, the physiological role and relation of vitamin D in glucose homeostasis can be studied in the cellular level and diabetic animal model of poor glycemic control.

## 5 Conclusion

Our study strongly demonstrates the inverse association of glycemic control as measured by HbA_1_c with the vitamin D status. Consequently, T2DM under poor glycemic control with vitamin D deficiency has a potential risk of dyslipidemia compared to those patients with insufficient and sufficient vitamin D. Thus, patients with T2DM who are at risk of developing diabetic complications under poor glycemic control should be advised for vitamin D measurement. This may help in subsequent vitamin D supplementation therapy and an increase in exposure to the sunlight, which may reduce the risk of CVD.
